# Care engagement with healthcare providers and symptom management self-efficacy in women living with HIV in China: secondary analysis of an intervention study

**DOI:** 10.1186/s12889-022-13573-3

**Published:** 2022-06-15

**Authors:** Wei-Ti Chen, Chengshi Shiu, Lin Zhang, Hongxin Zhao

**Affiliations:** 1 School of Nursing, University of California Los Angeles, 700 Tiverton Ave, Los Angeles, CA 90095 USA; 2 National Taiwan University, Taipei, Taiwan; 3 Shanghai Public Health Clinical Center, Fudan University, Shanghai, 201508 China; 4 Clinical and Research Center of Infectious Diseases, Beijing Ditan Hospital, Capital Medical University, Beijing, 100015 China

**Keywords:** Healthcare providers, HIV, Self-efficacy, Symptom management, Stigma, Women

## Abstract

**Background:**

Symptom management self-efficacy is a prerequisite for individuals to fully manage their symptoms. The literature reports associations between engagement with healthcare providers (HCPs), internalized stigma, and types of self-efficacy other than symptom management. However, the factors of symptom management self-efficacy are not well understood. This study aimed to investigate the relationship among engagement with HCPs, internalized stigma, and HIV symptom management self-efficacy in Chinese women living with HIV (WLWH).

**Methods:**

This current analysis was part of the original randomized control trial, we used data collected from 41 women living with HIV (WLWH) assigned to an intervention arm or a control arm from Shanghai and Beijing, China, at baseline, Week 4 and Week 12. The CONSORT checklist was used. The study was registered in the Clinical Trial Registry (#NCT03049332) on 10/02/2017.

**Results:**

The results demonstrate that HCPs should increase engagement with WLWH when providing care, thereby improving their symptom management self-efficacy. The results suggested that participants’ engagement with HCPs was significantly positively correlated with their HIV symptom management self-efficacy in the latter two time points. Internalized stigma was significantly negatively correlated with HIV symptom management self-efficacy only at the 4-week follow-up.

**Conclusions:**

This study demonstrated the positive effect of engagement with HCPs on WLWHs’ symptom management self-efficacy as well as the negative effect of internalized stigma on symptom management self-efficacy. Future research can further test the relationship between the three key concepts, as well as explore interventions to decrease internalized stigma.

## Introduction

As of 2018, there were approximately 850,000 people living with HIV (PLWH) in China [1]. Of those, about 28.6% were female [2]. The major transmission route for HIV among women in China is heterosexual contact, with the majority of infections happening outside the marital relationship [3]. Other transmission routes include blood selling and injection drug use [4]. In addition to female sex workers, housewives and career women have become infected by the virus [5, 6]. While trying to fulfill their family obligations, Chinese women living with HIV (WLWH) are also dealing with other challenges, including stigma, lack of financial and emotional support, and physical discomfort [7]. Since the development of antiretroviral therapy (ART), HIV infection can be managed as a chronic disease, but symptom management requires skills. Symptom management self-efficacy is a prerequisite for individuals to fully manage their symptoms. However, the potential factors influencing symptom management self-efficacy are not well understood. The literature reports associations among patients’ engagement with HCPs, perceived stigma, and other types of self-efficacy [8, 9]. For this study, we hypothesized that there are associations among patients’ engagement with HCPs, perceived stigma, and symptom management self-efficacy for WLWH in China.

## Background

Self-efficacy, which was conceptualized by Bandura (1986) and explicated in Social Cognitive Theory, is a well-recognized concept that contributes to behavior change [10]. Self-efficacy is said to be a person’s confidence in their ability to perform certain tasks regardless of difficulties or barriers [10, 11]. Huang et al. (2013) reported that HIV self-efficacy was positively correlated with quality of life among a sample of PLWH in China [12]. Other studies have demonstrated that PLWH who have high adherence self-efficacy can overcome side effects from ART as well as having better medication adherence [13]. Similarly, symptom management self-efficacy is a person’s confidence in conducting symptom-management related behaviors, which is a prerequisite for PLWH to adopt those behaviors [14]. Symptom management self-efficacy has been negatively associated with patients’ depressive symptoms, which means the better the symptom management self-efficacy, the fewer the depressive symptoms [15]. Also, symptom management self-efficacy has been reported to be a psychologically protective factor between the relationship of perceived stigma and quality of life among a group of PLWH in China [16].

For individuals with chronic diseases, such as PLWH, HCPs play an important role in supporting the management of their condition. Unlike other chronic diseases, HIV is highly stigmatized in certain populations and countries. Therefore, in these situations, HCPs might be the only people who can provide support for PLWH, which often results in PLWH maintaining care engagement with HCPs [17]. Evidence has shown that better engagement with HCPs was associated with various aspects of patient outcomes, including better mental health and quality of life [18], better medication adherence [9], and better care engagement [19].

Engagement with HCPs, one aspect of patient-provider relationships, is defined as an individual’s perception of their interaction with HCPs. Specifically, engagement with HCPs includes accessibility to and supportiveness of the providers, the patient’s involvement in healthcare decision making, and the level of mutual information sharing [20]. Studies have suggested that positive engagement with HCPs is critical for PLWH to develop effective self-management strategies [8, 21, 22]. In addition, engagement with HCPs was also associated with medication self-efficacy [23], medication adherence, and medical outcomes [20]. To date, there is a lack of studies that have focused on Chinese WLWH, especially regarding their engagement with HCPs.

Perceived HIV stigma is defined as the endorsement and application of negative feelings and beliefs related to HIV toward oneself [24, 25]. Studies have shown that perceived stigma is strongly associated with PLWH’s behavioral outcomes, including medication adherence and appointment attendance [24]. High levels of perceived stigma related to HIV has been frequently reported in Chinese PLWH populations [16, 26]. Stigma has led to PLWH in China having limited access to health care and other social resources, as well as to impacts on their behavioral outcomes and physical and mental health status [27, 28]. Stigma has been purported to negatively mediate the relationship among self-efficacy, medication adherence, and quality of life [26, 28]. In addition, a significant negative association has been found between perceived stigma and symptom management self-efficacy [16, 29].

Several studies have measured symptom management self-efficacy, engagement with HCPs, and perceived stigma separately [16, 26, 30]; however, limited empirical data is available on the relationships among these three factors, especially in WLWH. Therefore, this study investigates whether engagement with HCPs is associated with symptom management self-efficacy and whether these two factors are influenced by perceived stigma in Chinese WLWH. We hypothesized that better engagement with HCPs will improve symptom management self-efficacy and that higher perceived stigma will negatively affect symptom management self-efficacy.

## Methodology

### Study design, setting, and sampling

**Fig. 1:**
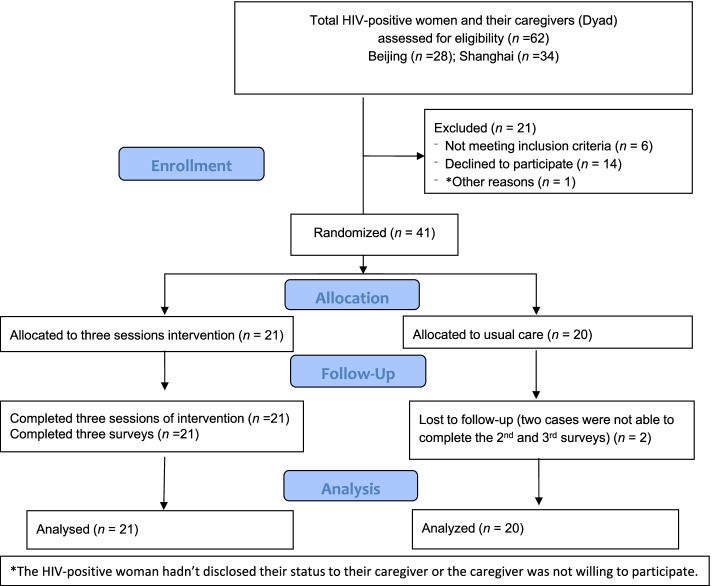
Consort Flow Chart

This current analysis was part of the original randomized control trial conducted in Beijing (Site 1) and in Shanghai (Site 2), China, from the summer of 2014 to the summer of 2016. In total, 41 WLWH were recruited for this study. This was a dyad analysis in which one WLWH and a family caregiver were considered a dyad; 62 dyads were screened, and total of 41 dyads (82 individuals: 41 WLWH and 41 family caregivers) consented to participate in the study. Then, 21 WLWH and a family member were assigned to the intervention group and 20 WLWH and a family member were assigned to the control group. The study hypothesis was that family members and WLWH who participated in the intervention would have better family support compared to the control group. However, in this article, the social support variable was not included in the analysis; therefore, the data from the family member was not included. The study was registered with the Clinical Trial Registry on 10/02/2017 (#NCT03049332). The CONSORT checklist was used in conducting the study; Fig. 1 presents a flow diagram of the study.

Inclusion criteria for participants in the study were (1) over 18 years old, (2) confirmed HIV seropositive, (3) at least one family member aware of the woman’s serostatus, (4) the family member was willing to participate in the study, and (5) literate in Chinese. Exclusion criteria included (1) cannot read/write in Chinese or communicate in Mandarin, (2) has not disclosed her serostatus to anyone, and (3) cannot complete the series of intervention sessions.

After securing their research consent, study participants were randomized to either the intervention or control arms. Three counseling sessions were delivered by nurse interventionists to the dyad participants (the woman living with HIV and her family member who was aware of her serostatus), over 4 weeks. Research staff reminded the dyad of the sessions via texts 2 days before and called 1 day before the encounter dates to ensure compliance. The intervention for self and family management consisted of five major components: family support; biofeedback for relaxation; cognitive–behavioral management skills; management of anxiety, stress, and depression; and psycho-education. Details of the intervention design, setting, and sampling were described in another paper [31]. The control group participants and their family caregivers were receiving the usual care, which included medication pick-ups, advice on the possible side effects of the medications, and conversations that WLWH and family caregivers originated with their physicians. All participants continued to receive the usual medical care at the clinical sites.

The U.S. team members trained two to four nurse interventionists at each site over one intensive week to ensure study fidelity. Also, nurse interventionists were supervised, and issues that arose during the counseling sessions were discussed by the nurse interventionists and the trainers via Skype and instant text messaging to maintain study fidelity. Three ACASI surveys were completed at baseline, Week 4, and Week 12 by all study dyads.

### Measurements

#### Demographic/background

Demographic information was collected, including age, education, income, and marital status. The information on participants’ HIV history was also collected, including the year of HIV diagnosis, ART status, possible infection route, CD4 count, and viral load.

#### Engagement with healthcare providers scale

The Engagement with Healthcare Providers scale is a 13-item measure rating clients’ perception of the nature of their interaction with their health care providers. The scale is a 4-point scale where 1 = *Always true* and 4 = *Never true*, and the total score ranges from 13 to 52, with a lower score indicating better HCP engagement. Cronbach’s alpha reliability estimate was 0.96 [20].

#### HIV symptom management self-efficacy

The HIV Symptom Management Self-Efficacy scale is a 10-item scale that assesses participants’ confidence in their ability to manage HIV-related symptoms. All items are rated on a 1–10 scale, where 1 = *Not at all confident* and 10 = *Totally confident*. A final score is calculated as the sum of all 10 items and ranges from 10 to 100. A higher score indicates better self-efficacy. The internal consistency reliability coefficient of the scale is 0.94 [15].

#### Perceived stigma

Berger’s HIV Stigma Scale [32],an eight-item instrument measuring internalization of stigma, was used. Questions measure the individual’s self-image, including a sense of uncleanliness, self-image (Does the subject perceive themselves as a bad person or inferior to others?), and a sense of shame and guilt. A 4-point Likert scale was used where 1 = *Strongly disagree* and 4 = *Strongly agree*. The sum of the eight items was calculated as the total score of perceived stigma. The score ranges from 8 to 32, with a higher score indicating a higher level of perceived stigma. The internal consistency of this scale was reported as 0.92 [33].

### Ethical considerations

The study protocol was reviewed and approved by three institutional review boards (IRBs) before participants were enrolled. The researchers explained the purpose and procedures of the study, answered questions, and obtained informed consent from participants before enrollment. In addition, the research staff expressed to all participants clearly that they had the right to withdraw from the study at any time without affecting their ongoing treatment at the study site. All data has been de-identified and kept in password-protected devices.

### Data collection

The research team at each site collected and managed their data independently. Audio Computer-Assisted Self-Interviews (ACASI) were conducted three times for each participant: at baseline, Week 4, and Week 12. All participants answered the study survey in Chinese, which was translated and back translated by four bilingual researchers; the study questionnaires have been used in previous studies in China with good reliability [9, 15, 23, 30, 31]. All the data, including the demographic data were collected using ACASI. The ACASI longitudinal data were later analyzed for publication.

### Data analysis

We first conducted descriptive analyses to understand our data. We inverted the score for the Engagement with Healthcare Providers scale, so a higher score reflects a better level of patient engagement with HCPs. Prior to performing the primary analysis with combined samples from two hospitals, we examined the equivalencies of sociodemographic characteristics between the two sites. We also calculated the intraclass correlation coefficient to measure the similarity of symptom management self-efficacy within each site. We calculated cross-sectional correlations among three primary outcomes (HCP engagement, symptom management self-efficacy, and internal stigma) at baseline, Week 4, and Week 12. We assessed the bivariate associations between each of the sociodemographic and HIV-related characteristics and the outcome variables using baseline data. We examined whether HCPs and perceived stigma affected symptom management self-efficacy over time using a mixed-effect model, which accounts for correlations within site and among subjects. We decided on a covariance structure for repeated data by comparing Akaike Information Criterion and Bayesian Information Criterion. The mixed models were performed with and without adjustment for years since diagnosis, which had substantive correlations with both symptom management self-efficacy and perceived stigma. Since the estimates of HCPs’ and perceived stigma effects did not change much with the adjustment, the final model did not include the covariate. Detailed values are presented in Table 3. All data analyses were performed using SAS Version 9.4.

### Findings

We collected and analyzed 122 questionnaires from 41 participants. At baseline, the average age of participants was 41.9 years (*SD* = 10.6; range = 21–61). Among the 41 participants, 39% (*n* = 16) had a high school or higher education, 51.2% (*n* = 21) had adequate income, and 73.2% (*n* = 30) were married or living with a partner. Also, 73.2% (*n* = 30) had been diagnosed with HIV for more than 1 year, and 82.9% (*n* = 34) were receiving ART. No statistical difference was identified between the Beijing and Shanghai sites (see Table 1).

**Table 1:** Baseline sociodemographic and HIV-related characteristics

	Total sample ***N*** = 41 ***M*** (***SD***) or ***N***(%)	Beijing ***N*** = 21 ***M*** (***SD***) or ***N***(%)	Shanghai ***N*** = 20 ***M*** (***SD***) or ***N***(%)	***P*** value
**Age**	41.9 (10.6)	42.7 (10.0)	41.0 (11.4)	.611
**Education**				
*≥ High school*	16 (39.0)	7 (33.3)	9 (45.0)	.444
**Income**				
*Adequate*	21 (51.2)	9 (42.9)	12 (60.0)	.272
**Marital status**				
*Married/having stable sexual partner*	30 (73.2)	15 (75.0)	15 (71.4)	.796
**Years since diagnosed** *> 1*	30 (73.2)	14 (66.7)	16 (84.2)	.201
**Current ART**				
*Yes*	34 (82.9)	17 (89.5)	17 (81.0)	.664
**Intervention group**				
*Yes*	20 (48.8)	10 (47.6)	10 (50.0)	.879

The mean score for engagement with HCPs was 37.7 (*SD* = 8.2) at baseline, a score that indicates greater engagement (possible range from 13 to 52). The average of these Chinese PLWH’s HIV symptom management self-efficacy scores at baseline was 65.1 (*SD* = 21.7), with a possible range from 10 to 100. Their mean perceived stigma score at baseline was 20.3 (SD = 3.5), with a possible range from 8 to 32. There was no significant difference in all three variables between the two sites.

Further analysis revealed that the mean scores for HIV symptom management self-efficacy increased slightly over time in both the intervention group (from 67.5 ± 21.7 at baseline to 71.4 ± 19.2 at the 3-month follow-up) and control group (from 62.9 ± 21.9 at baseline to 64.9 ± 22.9 at the 3-month follow-up). However, the increasing trend was sharper for the mean HIV symptom management self-efficacy scores of the Shanghai participants (from 66.0 ± 21.4 in baseline to 70.7 ± 18.4 at the 12-week follow-up) than that of the Beijing participants (from 64.3 ± 22.5 in baseline to 65.3 ± 23.9 at the 12-week follow-up) over time. Similarly, sharper increasing trends were identified for the mean scores of engagement with HCPs over time in the intervention group and at the Shanghai site. Mean perceived stigma scores did not change much either by intervention group or by site over time.

The results of bivariate correlation analyses (see Table 2) suggested that participants’ engagement with HCPs was significantly positively correlated with their HIV symptom management self-efficacy at the latter two time points. Perceived stigma was significantly negatively correlated with symptom management self-efficacy only at the 4-week follow-up. No statistically significant correlation was identified between the perceived stigma level and level of engagement with HCPs at any time point; however, the correlations were close to − 0.3, and these correlations had an insignificant effect on perceived stigma after adjusting for HCPs in our mixed effect models.

**Table 2:** Bivariate correlation by time for the three primary variables

	HIV Symptom Management Self-efficacy	Perceived Stigma
**Perceived Stigma**		
Baseline	−0.136	
Week 4	−0.376*	
Week 12	−0.153	
**HCPs Engagement**		
Baseline	−0.076	−0.298
Week 4	0.378*	−0.249
Week 12	0.411*	−0.272

*HCPs* Health care provider

* *p* < .05

Since we found a substantive cluster correlation (i.e., intraclass correlation coefficient) for the site, the longitudinal model (i.e., mixed-effect model) was adjusted for this correlation by adding a random effect of the site. We also examined the intervention effect on HIV symptom management self-efficacy by including a time-group interaction term in the model, but a significant intervention effect was not found. Table 3 shows the independent effects of the engagement with HCPs and perceived stigma and the adjusted effects of those in three mixed-effects models. In the independent models (i.e., Models 1 & 2), better engagement with HCPs (*p* = .029) and less perceived stigma (*p* = .021) were significantly associated with greater HIV symptom management self-efficacy over time. In the adjusted model (i.e., Model 3), neither of the predictors was statistically significant. However, the effect size of engagement with HCPs slightly decreased after adjusting for perceived stigma. The insignificant *p*-value for the association between engagement with HCPs might be due to the small sample size.

**Table 3:** Estimated effects of HCP and perceived stigma on Symptom management self-efficacy in mixed effect models

Independent Variables	Model 1 With HCP only	Model 2 With Perceived stigma only	Model 3 With HCP and perceived stigma
**HCP**	0.540 ± 0.243^*^	N/A	0.485 ± 0.249^* *^
**Perceived stigma**	N/A	−1.180 ± 0.504 ^*^	−0.633 ± 0.599
**Variance by Site**	49.079	24.647	51.863
**Variance by individual**	190.23	218.07	186.19
**Variance by residuals**	224.83	206.5	226.00

^*^. *p* < .05; ^* *^. *p* < .1

## Discussion

This study aimed to explore the relationship among WLWH’s engagement with HCPs, their perceived stigma level, and their HIV symptom management self-efficacy. Our results suggest that positive engagement with HCPs can independently predict better symptom management self-efficacy over time. Also, lower perceived stigma can independently predict better symptom management self-efficacy over time. These findings are well aligned with our hypothesis. In addition, we found that after putting both predictors into the model, engagement with HCPs has a stronger impact on symptom management self-efficacy than does perceived stigma.

Those WLWH with better engagement with HCPs were more likely to have better HIV symptom management self-efficacy. A similar relationship between engagement with HCPs and medication self-efficacy was reported in previous studies showing that positive engagement with HCPs enhanced participants’ confidence in their ability to adhere to their medication schedule [30, 34, 35]. This study confirmed that when WLWH have a more positive relationship with their HIV providers, they also have higher trust in self-care and more confidence in fighting the disease and managing HIV-related symptoms.

On the other hand, WLWH with higher perceived stigma are more likely to have a lower level of HIV symptom management self-efficacy. This finding was indicated by the binary analysis results, as the level of perceived stigma was negatively correlated with participants’ symptom management self-efficacy at the 4-week follow-up. Similar findings were reported in another group of Chinese PLWH, where perceived stigma negatively affected individuals’ symptom management self-efficacy, and symptom management self-efficacy buffered the negative impact of perceived stigma on PLWH’s quality of life [16]. Li and colleagues (2011) also reported a negative association between stigma and self-efficacy [35].

It is interesting to note that in the adjusted model with both predictors, engagement with HCPs remained a stronger influence on symptom management self-efficacy than did perceived stigma, even though none of the predictors were significant. We suspect collinearity between the two predictors, as we found a strong, though insignificant (due to the small sample size) correlation between the two predictors. No studies have been identified discussing the relationship between engagement with HCPs and perceived stigma or other types of stigma in general. Future studies are needed to investigate the relationship between the two factors, as well as how they interact to influence other cognitive, behavioral, and clinical outcomes.

This study was conducted among Chinese WLWH. Chinese WLWH react differently to symptom management self-efficacy than men because of their gender roles, as in this traditional culture, women tend to be more responsible for doing house chores, taking care of family members as well as making sure the ends meet [36, 37]. Also, WLWH are more likely to be victims of domestic violence and their household responsibilities are heavy, which can compromise their ability to cope with the disease [38]. Usually, they are the family caregivers for other family members, even if they are not feeling well. This added responsibility can compromise their medication adherence and disease outcomes [39]. Moreover, it is believed that WLWH perceive greater HIV-related stigma than do men [40]. Luckily, women respond to frequent patient-provider communication more positively than do men and more successfully achieve undetectable viral loads [40]. Therefore, it is of great importance for researchers to understand the effects of care engagement with HCPs in WLWH.

There are several limitations to this study. A small sample size with a convenience sample and each site only having about 41 WLWH hindered power calculations or stronger associations. Also, PLWH’s symptom management behaviors, self-management behaviors, and biomarkers (e.g., CD4 and viral load counts) were collected but not included in the analysis, which limited our understanding of the association between biobehavioral outcomes and symptom management self-efficacy directly. In addition, we recruited study participants from Beijing and Shanghai, where resources are more available compared to resource-limited areas in China, including limited access to healthcare providers. Therefore, the study results should not be generalized to all WLWH in China.

## Conclusion

This study demonstrated the positive effect of engagement with HCPs on WLWH’s symptom management self-efficacy, as well as the negative effect of perceived stigma on symptom management self-efficacy. The study contributes to understanding the role of engagement with HCPs in improving WLWH’s confidence in their ability to manage their symptoms and to improve their symptom-management skills. Future studies should test the level of influence that engagement with HCPs has on symptom-management behaviors directly. In addition, replicating this study on relationships among engagement with HCPs, perceived stigma, and symptom management self-efficacy in other HIV-affected regions and on other populations should be encouraged. Effective interventions to decrease perceived stigma should also be adopted.

### Relevance to clinical practice

HCPs should be aware that they can impact PLWH’s self-efficacy and, therefore, should give close attention to cultivating positive relationships with their patients living with HIV, especially Chinese women living with HIV who are willing to share their current difficulties with HCPs. Therefore, HCPs can reinforce the possible self- and family-management of these women and enhance their self-efficacy. In addition, interventions to decrease perceived stigma and enhance ART adherence among WLWH should be adopted.

## Data Availability

The datasets used and analyzed during the current study are available from the corresponding author upon request.
